# Characterization of extracellular vesicles released from *Prochlorococcus* MED4 at the steady state and under a light–dark cycle

**DOI:** 10.1098/rstb.2023.0339

**Published:** 2025-01-23

**Authors:** Ziqing Peng, Yaxin Liu, Haiying Ma, Shiwei Xiao, Allan Au-Yeung, Liang Zhang, Qinglu Zeng, Yusong Guo

**Affiliations:** ^1^ Division of Life Science and State Key Laboratory of Molecular Neuroscience, The Hong Kong University of Science and Technology, Hong Kong, China; ^2^ Department of Ocean Science, The Hong Kong University of Science and Technology, Hong Kong, China; ^3^ Department of Biomedical Sciences, and Tung Biomedical Sciences Center, City University of Hong Kong, Hong Kong, China; ^4^ Southern Marine Science and Engineering Guangdong Laboratory (Guangzhou), Guangzhou, 511458, China; ^5^ Key Laboratory of Biochip Technology, Biotech and Health Centre, Shenzhen Research Institute of City University of Hong Kong, Shenzhen, 518057, China; ^6^ Department of Precision Diagnostic and Therapeutic Technology, City University of Hong Kong Futian Research Institute, Shenzhen, China; ^7^ Hong Kong University of Science and Technology Shenzhen Research Institute, Shenzhen, China; ^8^ Thrust of Bioscience and Biomedical Engineering, Hong Kong University of Science and Technology, Guangzhou, China

**Keywords:** extracellular vesicles, *Prochlorococcus*, protein secretion, cargo sorting, diel cycle

## Abstract

Bacterial extracellular vesicles (EVs) are vesicles secreted by bacteria into the extracellular environment. Containing DNA, RNA and proteins, EVs are implicated to mediate intercellular communications. The marine cyanobacterium *Prochlorococcus*, the most abundant photosynthetic organism in marine ecosystems, has been shown to generate EVs continuously during cell growth. However, biogenesis and functions of EVs released by *Prochlorococcus* remain largely unclear. Here, we isolated and characterized EVs released by *Prochlorococcus* MED4 culture. We found that the majority of MED4 EVs are elliptical and enriched with specific proteins performing particular cellular functions. The light–dark cycle has been demonstrated to affect the cell cycle of *Prochlorococcus*, with cell division occurring at night time. Interestingly, we found that the net production of MED4 EVs was faster during the night time. Moreover, we revealed that MED4 EVs that are released or absorbed in the night time are enriched with distinct proteins, suggesting the release and absorbance of EVs are influenced by the diel cycle. We found that inhibiting cell division decreased the net production of MED4 EVs during the night time, suggesting that cell division is important for the biogenesis of MED4 EVs. These analyses provide novel insights into biogenesis and functions of EVs released from bacteria.

This article is part of the Theo Murphy meeting issue ‘Circadian rhythms in infection and immunity’.

## 1. Introduction

Extracellular vesicles (EVs) are vesicles released by cells to outer environments to play important roles in delivering lipids, proteins and nucleic acids from secreting cells to recipient cells. EVs have been demonstrated to be secreted by cells from the three domains of life, including eukaryotic, archaeal and bacterial cells [1]. In mammalian cells, EVs have been shown to shuttle mRNAs and change the gene expression of recipient cells, which are programmed to undergo differentiation or other cellular responses [2]. In immune systems, EVs are found to present antigens and stimulate immune cells [3]. Tumour-derived EVs are reported to play diverse roles in cancer development, including promoting metastasis and proliferation of cancer cells [4].

Unlike eukaryotic EVs, bacterial EVs have not been extensively investigated. Gram-positive and Gram-negative bacteria secrete EVs with different membrane structures, implying distinct mechanisms of EV biogenesis, which are still largely unknown [5]. More published studies focus on EVs secreted from Gram-negative bacteria, and they are also called outer-membrane vesicles (OMVs) [5]. Bacterial EVs show multiple functions in bacterial physiology and pathogenesis. EVs support the survival of bacteria in several ways, including serving as nitrogen sources and obtaining iron by iron acquisition proteins in EVs [6,7]. EVs protect bacteria from stress conditions by removing toxic compounds, absorbing antimicrobial peptides and interacting with bacteriophages [5,8].

In marine ecosystems, the unicellular Gram-negative cyanobacterium *Prochlorococcus* was the first autotroph described to produce EVs [6]. The high-light-adapted *Prochlorococcus* MED4 strain, isolated from the surface of the Mediterranean Sea, is the smallest phototroph, being 500–700 nm in diameter [9]. The tiny size means a high surface-to-volume ratio and thus efficient uptake of nutrients as a mechanism for *Prochlorococcus* MED4 to adapt to the nutrient-deprived surface seawater [10]. Presumably as another adaptive mechanism to survive in nutrient-deprived environments, *Prochlorococcus* strains release substantial amounts of EVs, reaching up to 10 times the number of cells in laboratory cultures [6]. EVs released by *Prochlorococcus* sustain the growth of heterotrophic bacteria and are shown to be physically associated with different marine microbes, indicating that *Prochlorococcus* EVs serve as an organic carbon source in marine food webs [6,11]. Negative stain transmission electron microscopy (TEM) revealed a direct interaction between EVs of *Prochlorococcus* MED4 and phages, suggesting that those EVs inhibit infection [6]. Analyses of lipidome, proteome and metabolome of vesicles released by *Prochlorococcus* MIT9312 and MIT9313 indicate EVs released from these two strains share some common compounds and also contain some strain-specific lipids, proteins and metabolites [11]. Although the proteome of MED4 EVs has been reported previously, the protein profiles were not extensively analysed and it remains unclear whether the identified cargo proteins are specially packaged and enriched in MED4 EVs [6]. To further explore functional roles of *Prochlorococcus* EVs, it is important to achieve a comprehensive view of their protein composition and perform quantitative analyses to uncover proteins specifically enriched in EVs.

Light is an essential environmental factor mediating the growth of *Prochlorococcus*. Typically, *Prochlorococcus* divide once per day and their cell division is synchronized to the daily light–dark (diel) cycle in natural environments or the artificial light–dark cycle in laboratory conditions [12,13]. For *Prochlorococcus* cells grown under light–dark cycles, DNA replication occurs in the afternoon, and cells divide in the night [13]. Light also influences gene expression of *Prochlorococcus* significantly, with 82% of expressed transcripts showing rhythmic patterns under light–dark cycles [14]. Many mammalian cells, such as tendon fibroblasts, maintain circadian rhythm in culture, and it has been reported that the protein composition of EVs released by tendon fibroblasts is influenced by the circadian clock [15]. Currently, it is unknown whether the light–dark cycle also affects the biogenesis and functions of EVs secreted from prokaryotic cells.

In this study, we isolated EVs released by *Prochlorococcus* MED4 and performed TEM and nanoparticle tracking analysis (NTA) to characterize the morphology, size distribution and concentration of MED4 EVs. Through label-free quantitative mass spectrometry (MS) analysis, we identified 23 proteins enriched in MED4 EVs, providing insights into the functional roles of MED4 EVs. In addition, we found that the relative production speed and protein compositions of MED4 EVs varied under light–dark cycles. Moreover, we detected that inhibiting cell cycle progression significantly reduced the abundance of MED4 EVs, indicating that cell division provides a mechanism for EV production in Gram-negative bacteria.

## 2. Results

### (a) Purification and characterization of extracellular vesicles released by *Prochlorococcus* MED4

**Figure 1.**
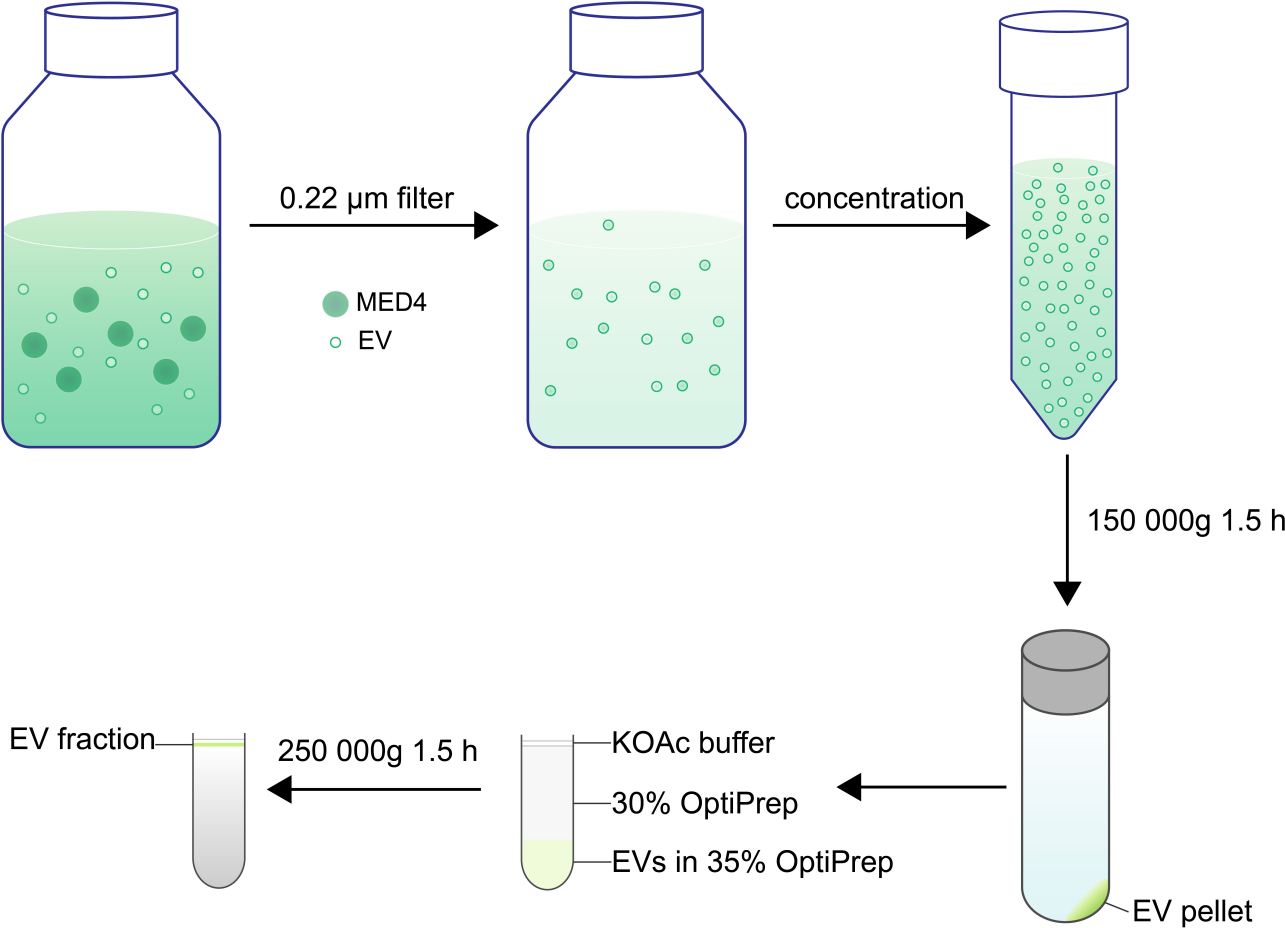
A diagram demonstrating the procedure to isolate extracellular vesicles (EVs) from *Prochlorococcus*. Batch cultures in the late exponential growth phase were filtered to remove MED4 cells. EVs were centrifuged by flotation through a two-step OptiPrep gradient. EVs are enriched at the interphase between the layer of 30% OptiPrep and the layer of buffer. Top fractions (EV fractions) containing enriched EVs were collected for further assays.

To isolate EVs released by *Prochlorococcus* MED4, we utilized an approach that is modified from a previously reported procedure for the same strain [6] (figure 1). Given that bacteria in the stationary phase undergo cell death and release cellular contents into the culture media, we chose to collect EVs when the cultures were in the log phase. We used 0.22 μm filters to remove MED4 cells (500−700 nm diameter) and carried out ultracentrifugation to pellet EVs. Flotation centrifugation was then performed to separate EVs from protein aggregates. Different from the previous method [6], the cell culture was only filtered once, to reduce the loss of EVs during the filtration step. Instead of loading several layers of OptiPrep with different concentrations, we used 35 and 30% OptiPrep layers [16], allowing EVs to become enriched at the junction between the 30% layer and the top layer. We used the potassium acetate (KOAc) buffer to preserve vesicle membranes that was often used to isolate vesicles [16]. After flotation, EVs were enriched in the top fraction (the EV fraction, figure 1) as described previously [16], with a density of 1.156 g ml^−1^, and enriched EVs were collected for the following assays.

**Figure 2.**
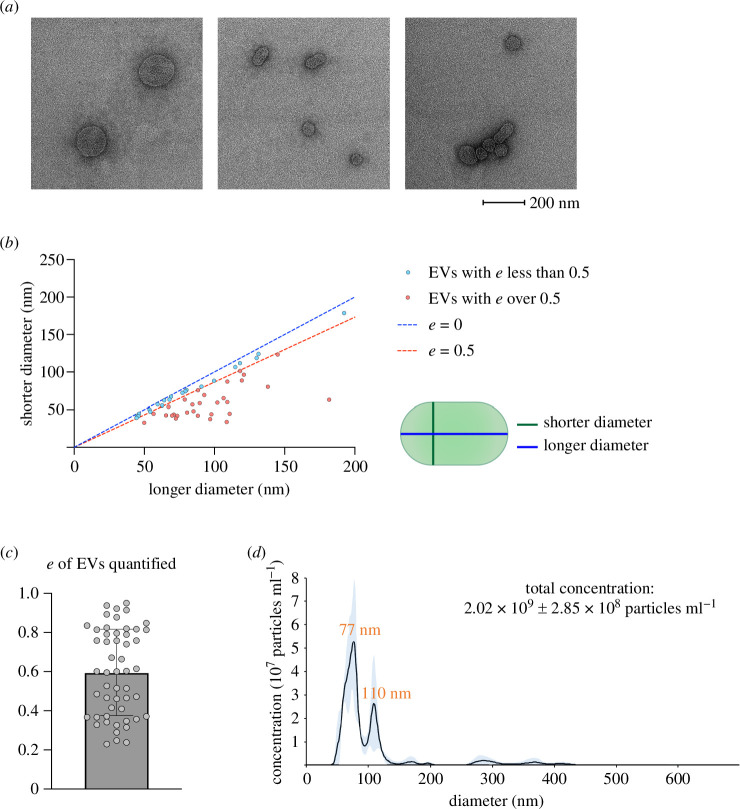
Characterizations of MED4 extracellular vesicles (EVs) by negative stain transmission electron microscopy (TEM) and nanoparticle tracking analysis (NTA). (*a*) Purified MED4 EVs detected by negative-stain TEM. (*b*) Quantification of the shorter and longer diameters of MED4 EVs based on TEM images of 52 vesicles purified from one experimental sample. EVs are divided into two groups based on eccentricity (*e-value*). Red dots represent EVs with *e*-values higher than 0.5, while blue dots represent the rest of the MED4 EVs. (*c*) A bar graph presenting the average of the *e*-values calculated (mean ± s.d.; *n* = 52) for MED4 EVs. Each dot represents an individual MED4 EV. (*d*) Size distributions and concentrations of MED4 EVs analysed by NTA. Blue area represents 95% confidence interval based on three technical repeats.

To characterize the morphology of *Prochlorococcus* MED4 EVs, we visualized them by negative stain TEM. We detected numerous membrane structures in the EV fraction (figure 2*a*), noticing that some MED4 EVs showed elliptical shapes. Such elliptical membrane structures were not observed in vesicles of mammalian cells isolated using similar experimental procedures [16]. To define elliptical EVs, we used eccentricity (referred to as *e*) as a parameter (see §4). The *e*-value ranges from 0 to less than 1 (0 ≤ *e *< 1). An EV with an *e*-value of 0 represents a perfect sphere, while a value of 0.5 or greater indicates a more elliptical or even rod-like shape [17]. We quantified the *e-*values of 52 EVs and found that more than half of these EVs exhibited an eccentricity exceeding 0.5, with a mean value close to 0.6 (figure 2*b*,c). Nanoparticle tracking analysis (NTA) of the EV fraction indicates that the size distribution of MED4 EVs contains two peaks: 77 and 110 nm (figure 2*d*). Using NTA, we calculated that purified MED4 EVs reached 3.3 EVs per cell, consistent with the previous report using the same strain [6].

### (b) Proteomic analysis of extracellular vesicles secreted from *Prochlorococcus* MED4

**Figure 3.**
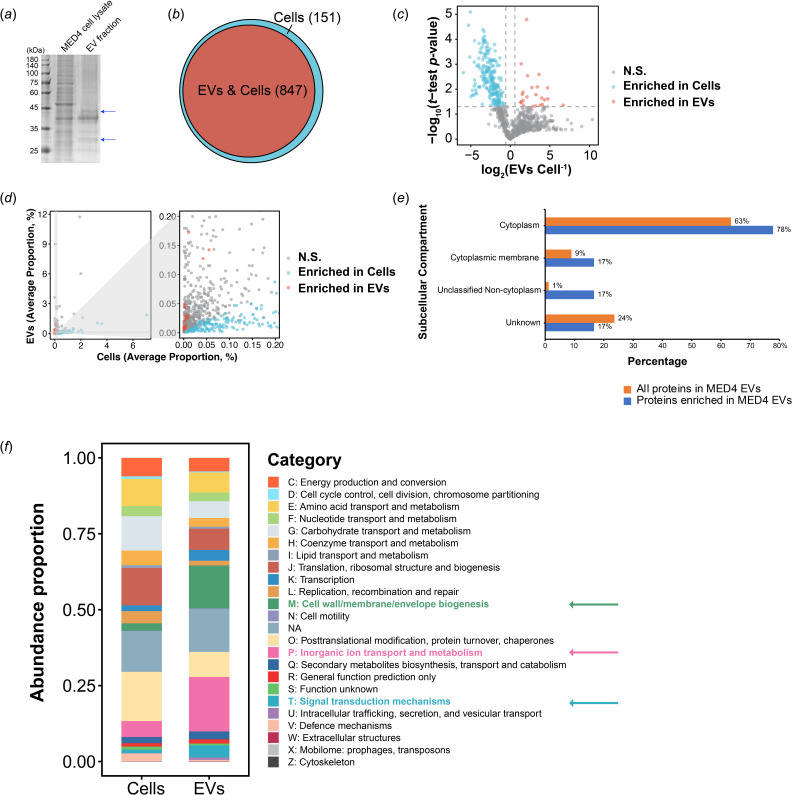
Label-free quantitative proteomic analysis of the protein composition of MED4 extracellular vesicles (EVs). (*a*) Coomassie blue staining to analyse the proteins in MED4 cell lysates and MED4 EVs. (*b*) Venn diagram showing the number of shared (orange) and unique proteins identified in MED4 cell lysate (blue). (*c*) Volcano plot of shared proteins in three biological repeats. Based on the proportion of each identified protein, the mean log_2_(fold change) and -log_10_(*p*‐value) were calculated and plotted on the *x*-axis and *y*-axis, respectively. The identified proteins with log_2_(EVs cell^−1^) >0.58 and *p*‐value <0.05 are considered to be enriched in EVs (orange dots) and proteins with log_2_(EVs cell^−1^) <−0.58 and *p*‐value <0.05, are considered to be enriched in cell lysates (blue dots). N.S. represents proteins without significant differences of average abundance in cells and EVs. (*d*) Scatter plot of proteins identified in EVs. The *x*-axis indicates average proportion in cells while the *y*-axis indicates average proportion in EVs. The right panel shows higher magnification of the area highlighted in grey in the left panel. N.S. represents proteins without significant differences of average abundance in cells and EVs. (*e*) Percentages of all proteins identified in MED4 EVs and proteins identified to be enriched in MED4 EVs that are predicted to be located at the indicated subcellular localizations. (*f*) The proportions of Clusters of Orthologous Groups of proteins (COG) functional categories in proteins identified in cell lysates and proteins identified in EVs based on protein abundance.

To investigate the protein profilings of MED4 cells and EVs, we performed proteomic analysis of MED4 EVs produced under continuous light, under which condition *Prochlorococcus* cells were not synchronized and thus were at different stages of the cell cycle. Coomassie blue staining analysis indicated that MED4 cell lysates and MED4 EVs have different protein compositions (figure 3*a*). Some apparent bands in the EV sample were not obviously detected in the cell lysates (arrows in figure 3*a*), suggesting that EVs were enriched with a specific group of proteins. To reveal proteins that are specifically enriched in MED4 EVs, we performed in-solution digestion with trypsin followed by label-free MS analysis. In MED4 cell lysates and EVs, we identified 998 proteins in total (electronic supplementary material, table S1, sheet 1), of which 151 were distinct in cell lysates and 847 were shared between both groups (figure 3*b*). For the shared proteins, we calculated the proportion of each protein in cell lysates or in EVs, respectively, based on the normalized abundance of each identified protein (see §4). The average fold change of the proportion of a specific protein in the EVs group divided by the proportion of this protein in the cell lysate group was quantified based on three biological repeats. The *p*-value was quantified based on the proportion in the EV group and in the cell lysate group in three biological repeats. A volcano plot of the log_2_(fold change) (*x*-axis) versus log_10_(*p*-value) (*y*-axis) was generated (figure 3*c*). We identified 23 enriched proteins in MED4 EVs (orange dots in figure 3*c*; electronic supplementary material, table S1, sheet 2) and 217 enriched proteins in cell lysates (blue dots in figure 3*c*; electronic supplementary material, table S1, sheet 3). In addition, for proteins enriched in EVs, their proportions in cell lysates were low and mainly close to 0 (figure 3*d*). For proteins enriched in cell lysates

, their proportions in EVs were mainly between 0 and 0.05% (figure 3*d*). This difference of enrichment suggested that the 23 proteins we identified were specifically packaged in EVs, and this enrichment process is presumably mediated by some unknown cargo sorting machinery.

A previous study based on in-gel digestion identified 40 proteins in MED4 EVs [6]. Owing to the development of MS technology, we identified 20 times more proteins in MED4 EVs with in-solution digestion (847 proteins), 25 of which were also found in the previous study [6] (electronic supplementary material, table S1, sheet 4). In addition to *Prochlorococcus* MED4, label-free quantitative MS analysis identified 111 proteins shared by EVs released from *Prochlorococcus* strains MIT9312 and MIT9313 [11]. We detected 149 proteins in both MED4 and MIT9313 EVs (electronic supplementary material, table S1, sheet 5), and 201 proteins in both MED4 and MIT9312 EVs (electronic supplementary material, table S1, sheet 6), and 87 proteins were shared by these three strains (electronic supplementary material, table S1, sheet 7).

We predicted the subcellular compartment of MED4 EV proteins (847) as well as the EV-enriched proteins (23) among these 847 EV proteins following a previous protocol [11] (electronic supplementary material, table S1, sheet 8). We found that a significant portion of MED4 EV proteins and MED4 proteins that are specifically packaged in EVs came from the cytoplasm, reaching 63 and 78%, respectively (figure 3*e*). In addition, the percentage of EV-enriched proteins predicted to be localized at the membrane (inner membrane and thylakoid membrane) was more than that detected in all MED4 EV proteins (figure 3*e*). We then performed Clusters of Orthologous Groups of proteins (COG) analysis to analyse the function of all proteins identified in cell lysates and EVs (figure 3*f*). Compared with cell lysates, EVs contained a higher relative abundance of proteins involved in some categories (e.g. cell wall/membrane/envelope biogenesis, inorganic ion transport and metabolism, signal transduction mechanisms) and a lower relative abundance of proteins in some categories (e.g. translation, ribosomal structure and biogenesis, replication, recombination and repair, posttranslational modification, protein turnover, chaperones). Among the 23 proteins enriched in EVs, five were associated with ‘hydrolase activity’, including three proteinases; five were linked to ‘ion binding’; and two were associated with ‘transporter activity’ according to their GO annotations (electronic supplementary material, table S1, sheet 2). Our data are consistent with the previous conclusion that the functions of EVs may be related to the hydrolysis of macromolecules [5].

### (c) MED4 extracellular vesicle production was enhanced in the dark phase compared with the light phase

**Figure 4.**
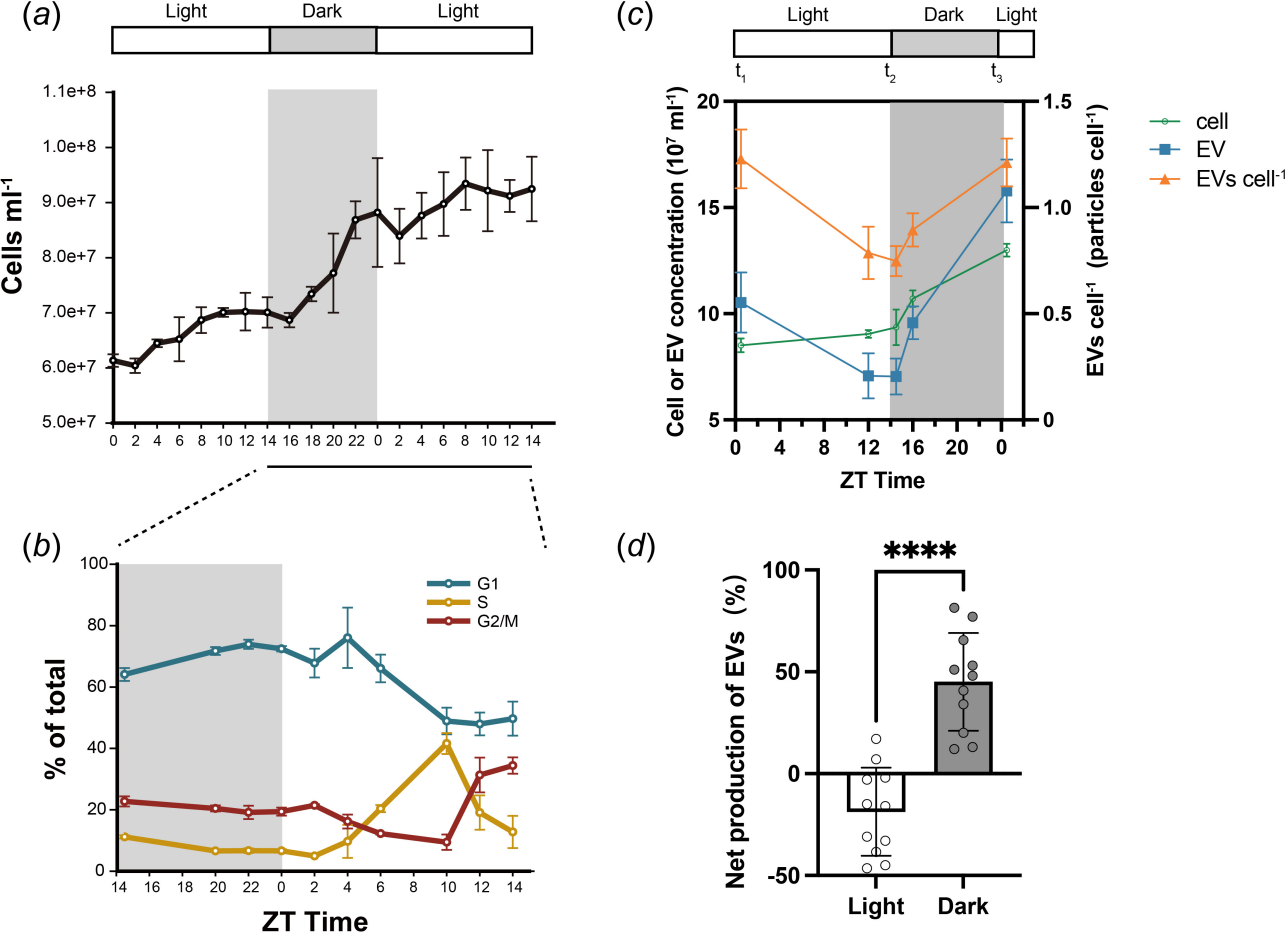
The secretion of MED4 extracellular vesicles (EVs) under light–dark cycles. (*a*, *b*) Population growth (*a*) and cell cycle (*b*) of synchronized *Prochlorococcus* MED4. Batch cultures of *Prochlorococcus* MED4 were grown under 14 h : 10 h light–dark cycles for at least three months before the assay. The period without illumination, from zeitgeber time (ZT) 14 to 24 is coloured in grey. Error bars represent the standard deviations from three biological replicates. (*c*) Line chart of the cell concentration, EV concentration and relative concentration of EVs (EVs cell^−1^) during 24 h. EVs were collected by filtering through 0.22 μm filters at ZT 0.5, 12, 14.5, 16 and ZT 0.5 in a new cycle, followed by purification. Quantification was based on three representative biological replicates (mean ± s.d.). (*d*) Bar graph of the net production of EVs under diel cycles from 11 biological repeats (mean ± s.d.; *n* = 11; paired *t*‐test, ****p < 0.0001). For each 24 h, we used *t*_1_ -*t*_3_ (highlighted in (*c*)) to represent EVs er cell for each time point chronologically. The relative production of EVs in the light phase = (*t*_2_ − *t*_1_)/*t*_1_ and that in the dark phase = (*t*_3_ − *t*_2_)/*t*_2_.

Next we analysed whether EV production rate of *Prochlorococcus* cells changes under light–dark cycles. The population growth and cell cycle of *Prochlorococcus* MED4 synchronized to 14 h : 10 h light–dark cycles were analysed by flow cytometry (figure 4*a*). The concentration of cells mainly increased at night (figure 4*a*). The proportions of cells in the G_1_/S and G_2_/M phases also suggested a synchronized cell cycle of *Prochlorococcus* MED4 (figure 4*b*) [13]. To investigate the quantity of EVs under a light–dark cycle, we analysed cell and EV concentrations, as well as the relative concentrations of EVs per cell from synchronized MED4 culture. Samples were collected at the beginning and end of both the light and dark phases (zeitgeber time (ZT) 0.5, 14.5, and again at ZT 0.5 in a new cycle), as well as one additional time during each phase (ZT 12 and 16) (figure 4*c*), with EVs purified as previously described (figure 1). Our results showed that the relative concentration of EVs per cell decreased during the light phase but increased during the dark phase (figure 4*c*). Notably, during the night phase, both cells and EVs exhibited an increase, with a more pronounced rise in EVs, leading to an increased ratio of EVs per cell (figure 4*c*). Consequently, the net production of EVs was significantly higher in the dark phase compared with the light phase (figure 4*d*).

The net production of EVs is determined by both EV and cell concentrations. EV concentration is influenced by the speed of the secretion of EVs and the speed of the uptake of EVs. In addition, degradation, aggregation or fusion of EVs can also affect the EV concentration. Cell concentration is influenced by cell growth and cell death. It has been reported that the size and concentration of EVs released by *Prochlorococcus* are stable over the course of 2 weeks in sterile seawater [6], suggesting that the loss of *Prochlorococcus* EVs caused by degradation, aggregation or fusion is minimal. Thus, we hypothesize that EV secretion and EV uptake are the two major factors that determine the net production of EV. We found that the net production of EVs in the light phase was lower than 0, indicating uptake speed was higher than secretion speed during the daytime. In contrast, the speed of EV secretion is faster than the speed of EV uptake during the night time.

### (d) MED4 extracellular vesicles that are released or absorbed in the night time are enriched with distinct groups of proteins

**Figure 5.**
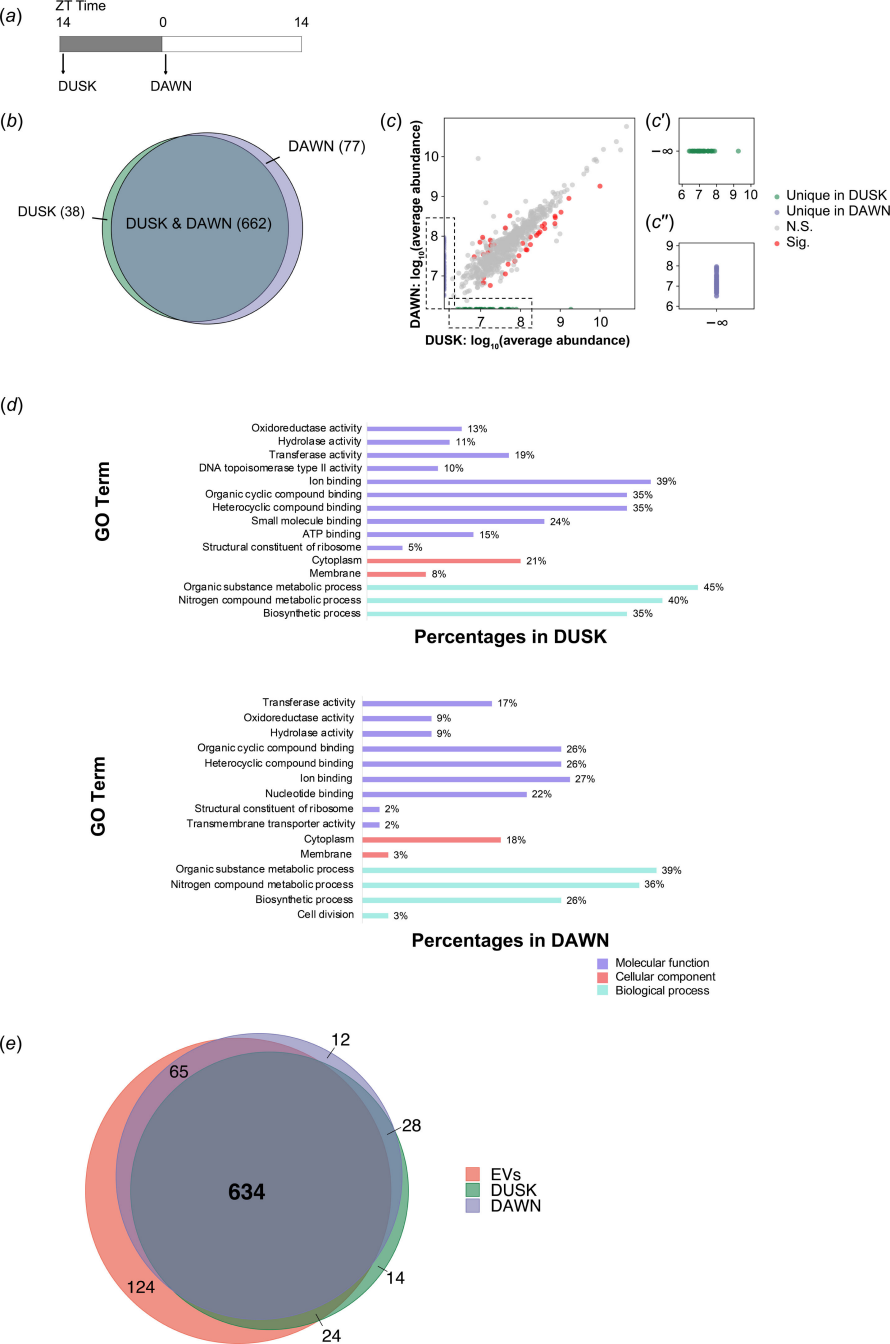
Label-free quantitative proteomic analysis of the protein composition of MED4 extracellular vesicles (EVs) secreted at the start of the dark or light phase. (*a*) Time bar of EV collection in a dark phase (indicated by arrows). Grey area indicates the dark phase while white area indicates the light phase. Batch cultures of *Prochlorococcus* MED4 were grown under a 14 h light : 10 h dark cycle for at least 3 months before the assay. EVs were collected by filtering through 0.22 μm filters at ZT 14.5 (DUSK) and ZT 0.5 (DAWN), followed by purification. (*b*) Venn diagram showing the number of shared (light blue) and unique proteins identified in DUSK (green) and DAWN (purple). (*c*) Scatter plot of proteins identified in EVs secreted by synchronized MED4. The *x*-axis indicates log_10_(average abundance in DUSK) and *y*-axis indicates log_10_(average abundance in DAWN). (c′,c″). Magnified views of area indicated by the dotted boxes in (*c*). N.S., proteins without significant differences of average abundance in DUSK and DAWN. Sig., proteins significantly enriched in DUSK or DAWN. (*d*) Gene ontology (GO) analysis of proteins uniquely identified or enriched in DUSK or DAWN. Values next to the bars represent percentages of proteins identified in specific categories. (*e*) Comparison of the identified MED4 EV proteins secreted under continuous light or secreted at DUSK or DAWN.

Our analyses indicate that the speed of EV secretion is higher than the speed of EV uptake in the dark phase. Are EVs taken up in the dark phase and are EVs released in the dark phase enriched with different groups of proteins? To investigate this question, we isolated MED4 EVs only from two successive time points across one night (figure 5*a*, DUSK and DAWN), then performed label-free MS. In this case, DUSK samples contained EVs released during the day time. We propose that proteins whose abundances are significantly enhanced in the DAWN group are those that are enriched in MED4 EVs secreted in the night time. We also propose that proteins whose abundances are significantly decreased in the DAWN group are those that are enriched in MED4 EVs absorbed in the night time. We identified 777 proteins in total (electronic supplementary material, table S2, sheet 1). Among them, 38 proteins were uniquely identified in DUSK, while 77 proteins were uniquely identified in DAWN (figure 5*b*,*c*; electronic supplementary material, table S2, sheets 2 and 3). We then performed protein enrichment analysis utilizing quantification methods described previously. This analysis revealed that 627 proteins showed a similar enrichment in the EVs isolated from the two experimental groups (DUSK and DAWN) and 35 showed a different enrichment (figure 5*c*). Among these 35 proteins, 24 proteins were found to be more enriched in DUSK, while 11 proteins were found to be more enriched in DAWN (electronic supplementary material, table S2, sheets 4 and 5). These analyses uncovered EVs enriched with specific proteins that are secreted in the night time (unique or more enriched in DAWN), and also identified EVs enriched with specific proteins that are absorbed in the night time (unique or more enriched in DUSK).

In total, 150 proteins were uniquely identified or enriched in DUSK or DAWN. A previous study, using RNA-sequencing and MS, identified 312 genes exhibiting diel cycling patterns in cells [18]. Upon comparison, 21% (31 out of 150) of these proteins showed diel oscillations in their intracellular levels. Among these, three proteins maintained a consistent abundance pattern in both EVs and cells, whereas 10 proteins showed opposite trends in abundance changes. The remaining 18 proteins peaked in abundance during the middle of the light or dark phase and did not exhibit significant changes at the two specific time points sampled in our study. For most proteins that displayed significant differences in abundance within DUSK or DAWN EVs, their enrichment appeared to be independent of the corresponding protein levels in the cells at those respective time points.

We performed gene ontology (GO) analysis of these 150 proteins uniquely identified or with significant enrichment in DUSK or DAWN (figure 5*d*). In both DUSK and DAWN, numerous identified proteins were associated with catalytic activity and ion binding; these were also main annotations of proteins enriched in MED4 EVs (electronic supplementary material, table S2). Eighty-five per cent of EV proteins identified under continuous light were also found under a light–dark cycle (figure 5*e*), suggesting the consistency of our proteomic analysis.

Among unique proteins found in DAWN, we found two proteins associated with membrane biogenesis. One of them was UDP-3-*O*-acyl-glucosamine *N*-acyltransferase (LpxD), an important transferase for the biosynthesis of outer membrane [19]. Another was UPD-*N*-acetylmuramoylalanine-d-glutamate ligase (MurD), involved in the biogenesis of peptidoglycan to form the cell wall [20]. The presence of these proteins suggests active membrane synthesis in the dark phase, and we hypothesize that it was one possible reason why MED4 secreted EVs faster in the dark phase.

In addition, we found the circadian clock protein KaiB among the top abundant proteins uniquely identified in the proteome of EVs produced at night. In *Prochlorococcus*, KaiB and KaiC make up the central timing system. In *Synechococcus*, 50–80% of KaiB protein was found in the membrane fraction [21], which is consistent with the existence of KaiB in membrane vesicles. Moreover, the abundances of both total cellular and membrane KaiB peaked at approximately circadian time (CT) 16 [21], which was in the subjective night. We hypothesize that the enhanced membrane localization and the increased EV production at night contribute to the high abundance of KaiB in EVs produced at night.

### (e) Cell division regulates the biogenesis of MED4 extracellular vesicles

**Figure 6.**
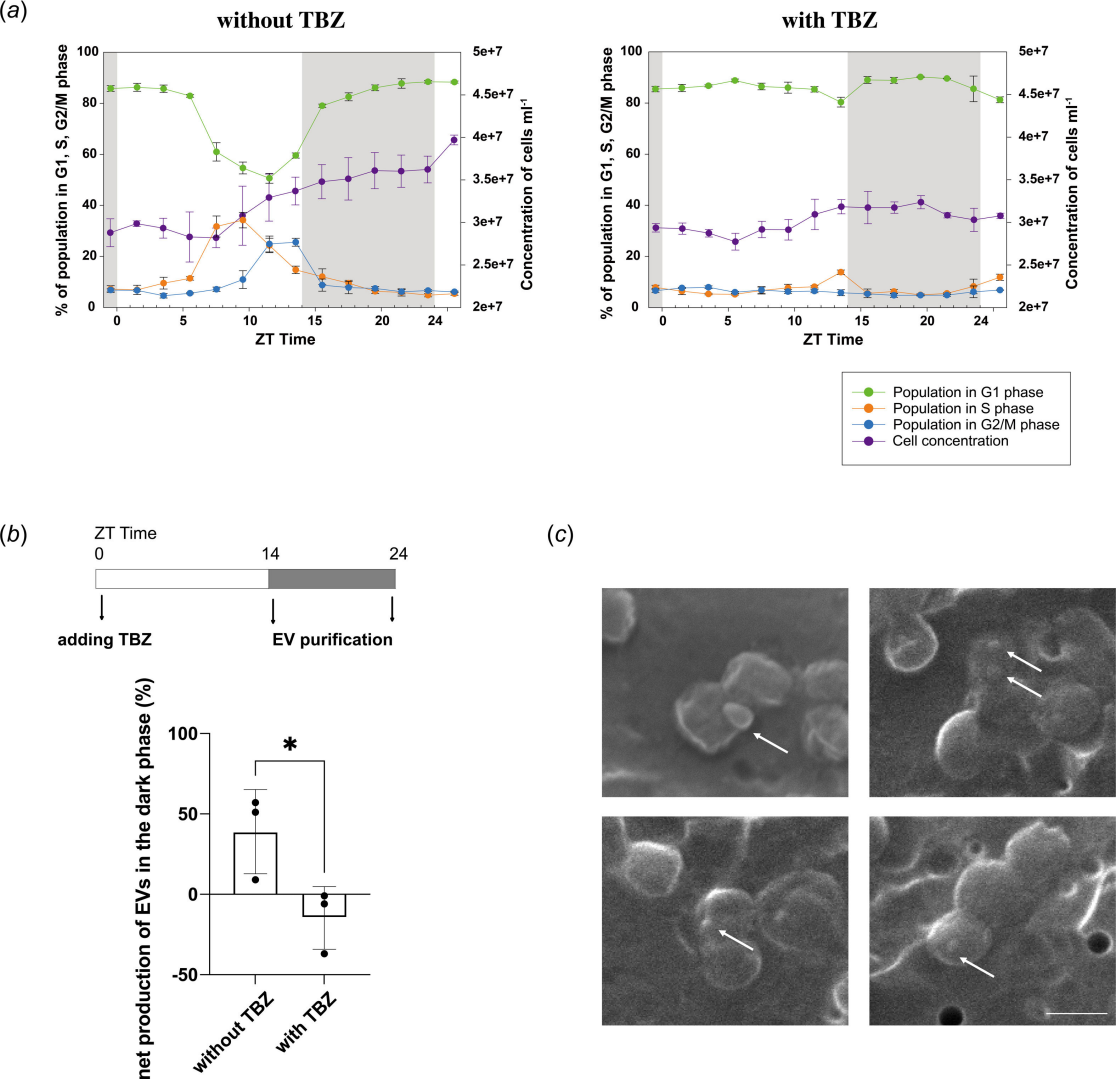
Cell division regulates the biogenesis of MED4 extracellular vesicles (EVs). (*a*) Cell cycle of MED4 with or without thiabendazol (TBZ) treatment. The period without illumination, from zeitgeber time (ZT) 14 to 24 is coloured in grey. Error bars represent the standard deviations from three biological replicates. (*b*) Quantification of net production of EVs in the dark phase with or without TBZ treatment from three biological repeats (mean ± s.d.; *p* < 0.05). (*c*) Scanning electron microscopy images of MED4 during cell division. Cells were collected at ZT 14 without TBZ treatment. EVs are indicated by white arrows. Scale bar, 500 nm.

Our results revealed an increased net production of MED4 EVs in the dark phase, which is accompanied by enrichment of proteins involved in membrane biogenesis. As the cell division of *Prochlorococcus* was mostly completed in the dark phase [12] (figure 4*a*), we tested whether the net production of EVs would be inhibited when cell division was blocked. Thiabendazole (TBZ), a tubulin assembly inhibitor, has been used to inhibit the cell division of cyanobacteria [22]. We applied it to induce cell cycle arrest of *Prochlorococcus* MED4 by adding TBZ at ZT 0.5. As expected, TBZ treatment prevented cells from entering S phase and cell numbers were stable, while in the control group (without TBZ) cells divided and cell numbers increased (figure 6*a*). We added TBZ at ZT 0.5 and purified EVs at ZT 14.5 and 23.5. The net production of EVs was detected to be increased in the dark phase without TBZ treatment (figure 6*b*), consistent with previous analysis (figure 4*c*,*d*). In contrast, the net production of EVs in the dark phase was significantly reduced with TBZ treatment (figure 6*b*). In addition, we observed nascent EVs budding from dividing MED4 cells by scanning electron microscopy (SEM) (figure 6*c*). These analyses support that biogenesis of MED4 EVs is regulated by cell division.

## 3. Discussion

In this study, we isolated MED4 EVs through biochemical approaches and analysed the protein profiling of MED4 EVs through label-free quantitative MS. We identified 23 proteins that were more enriched in EVs than in cell lysates, though the mechanisms driving their specific enrichment are not yet understood. We suggest that these proteins are actively sorted into the vesicles, rather than being randomly packaged. Many of these 23 proteins are transporters and hydrolases, providing insight into functional roles of MED4 EVs. We hypothesize that the transporters enriched in EVs bind metal ions in the extracellular environment, and uptake of EVs by MED4 or other microbes allows microbes to acquire metal. The hydrolases enriched in MED4 EVs may function to degrade macromolecules to small molecules for the microbes to absorb. Further analyses need to be performed to elucidate the functional roles and fates of MED4 EVs.

The morphology of EVs, typically observed through cryo-electron microscopy, generally presents as spherical or cup-shaped. However, in our study, we found that the majority of MED4 EVs exhibit an elliptical shape. This observation is consistent with a previous report, demonstrating the presence of rod-like or tubular EVs in various cell types [17]. The persistence of these elliptical shapes in EVs could be influenced by several factors, such as the specific lipid or protein composition of the membrane, the presence of curvature-inducing proteins, and cytoskeletal components.

Budding of EVs was captured in actively dividing Gram-negative bacteria, *Salmonella*, and the budding events were shown to take place in division septa and the cell body [23]. TEM images revealed that EVs released from division septa are larger while EVs released from the cell body are smaller [23]. These analyses indicate that the cell cycle progression may influence the biogenesis of EVs released by *Salmonella*. The cell cycle of cyanobacteria is regulated by the diurnal rhythm. Here, we found that the light–dark cycle influences EV production. Moreover, inhibiting cell division significantly reduced the net production of EVs in the dark phase. Based on these observations, we hypothesize that division of MED4 promotes MED4 EV production in the dark phase.

The Endosomal Sorting Complex Required for Transport (ESCRT) is an evolutionarily conserved machinery that plays critical roles in the biogenesis of EVs [24]. This machinery comprises four complexes (ESCRT-0, -I, -II, and -III) assembled from nearly 30 proteins, and they are named from the order in which they function in the yeast ESCRT pathway [25]. ESCRT drives the formation of multivesicular bodies (MVBs), necessary in EV production of eukaryotes [25]. In archaea, EV biogenesis was also reported to depend on ESCRT [26]. Among the ESCRT family, ESCRT-III participates in membrane scission [24], and the expression of ESCRT-III homologues in archaea is linked to cell division [27]. Proteins in the ESCRT family were also found in bacteria. As an example, PspA, an ESCRT-III-like protein in *Synechococcus*, remodels bacterial membrane followed by vesicle growth and cross-membrane vesicle transfer [26]. Our study reveals that cell division regulates biogenesis of *Prochlorococcus* EVs. ESCRT machinery is important for cell division in archaea and eukaryotic cells [28], but whether ESCRT proteins are involved in bacterial cell division and EV secretion remains uncertain.

*Prochlorococcus* EVs are shown to be taken up by heterotrophs and they are implicated in providing nutrients to support the growth of heterotrophs [6]. In low-nitrogen environments, *Prochlorococcus* was reported to outgrow its rival *Synechococcus* only in the presence of heterotroph *Alteromonas macleodii* [29]. As one of the components in *Prochlorococcus* exudate, EVs may play essential roles in the interaction between cyanobacteria and heterotrophs. Diel EV production of *Prochlorococcus* can provide diel nutrient supply in the marine environment, thereby affecting the growth of heterotrophs.

## 4. Material and methods

### (a) The growth of *Prochlorococcus*

Axenic *Prochlorococcus* MED4 was grown in filtered and autoclaved Port Shelter (Hong Kong) seawater-based Pro99 medium [30]. Axenic tests were performed each month [30]. To characterize EVs released from unsynchronized MED4, batch cultures were maintained under constant light (25 μmol photons m^−2^ s^−1^) at 23℃. To investigate MED4 EVs released under a diel cycle, batch cultures were maintained under 14 h : 10 h light–dark cycles (100 μmol photons m^−2^ s^−1^ in the light phase) at 23℃ for more than 3 months before related assays. The population growth and cell cycle phase of *Prochlorococcus* were measured by flow cytometry, following a published protocol [14]. In brief, preserved *Prochlorococcus* cells were stained with SYBR green (Invitrogen). Stained cells were analysed by an Accuri C6 Plus flow cytometer (BD Biosciences) with the BD CSampler Plus software. Cell cycle phases were determined by the FlowJo V10 software (BD Biosciences).

### (b) Collection of *Prochlorococcus* MED4 extracellular vesicles before purification

For proteomic analysis, batch *Prochlorococcus* cultures were grown to late exponential growth phase in order to collect a large number of EVs. Over 500 ml culture was used in one proteomic sample.

To measure the concentration of MED4 EVs under light–dark cycles, batch *Prochlorococcus* cultures in exponential phase were used. For each sample, 20 ml culture was used. To test whether MED4 EVs production rate changes under light–dark cycles, batch cultures were filtered to remove cells at the beginning and the end of light or dark phase (ZT 0.5 and ZT 14.5). To test the effect of cell division inhibitor TBZ on MED4 EV generation, we added TBZ (Sigma-Aldrich, 30 μg ml^−1^ stock in Pro99) or Pro99 medium into batch cultures at ZT 0.5. Batch cultures were filtered to remove cells at ZT 14.5 and ZT 23.5. The filtrate was stored at 4℃ before EV purification.

### (c) Purification of *Prochlorococcus* MED4 extracellular vesicles

*Prochlorococcus* MED4 EVs were purified with a modified protocol based on published procedures [6,31]. For cultures over 500 ml, we concentrated the filtrate by tangential flow and the volume was reduced to 40−160 ml. EVs and protein aggregates were sedimented after ultracentrifugation for 1.5 h at 150 000*g* at 4℃ (Hitachi CP80WX Preparative Ultracentrifuge, rotor T865 or SW41 Ti). We resuspended the pellets with 400 μl 40% (v/v) OptiPrep in KOAc buffer (110 mM potassium acetate, 20 mM 4-(2-hydroxyethyl)piperazine-1-ethane-sulfonic acid (HEPES) and 2 mM magnesium acetate, pH 7.2) and overlaid 800 μl 30% (v/v) OptiPrep on the resuspended fraction and 50 μl KOAc buffer only as the top fraction. The gradients were then centrifuged in a swing-out rotor (Hitachi CS150NX Micro Ultracentrifuge, rotor S55S) for 1.5 h at 250 000*g* at 4℃. After flotation, the top 200 μl was collected for transmission electron microscopy (TEM) analysis and nanoparticle tracking analysis(NTA). For Coomassie blue staining and MS analysis, EVs were pelleted by ultracentrifugation for 30 min at 120 000*g* at 4℃ (Hitachi CS150NX Micro Ultracentrifuge, rotor S120-AT3) after diluting with 300 μl KOAc buffer.

### (d) Transmission electron microscopy analysis

For TEM analysis, 5 μl EV fraction was loaded on a charged copper grid and left for 30 s. Then the copper grids were washed with double-distilled H_2_O two times and 2% (w/v) uranyl acetate one time. The copper grids with EVs were stained with 2% (w/v) uranyl acetate for 30 s. After air drying for 2 min, the stained copper grids were imaged under a Talos transmission electron microscope at 120 kV. To calculate the eccentricity value (*e*) for each EV, we employed the following equation based on the longer diameter (*a*) and shorter diameter (*b*): *e* = $\sqrt{1-b²/a²}$.

### (e) Scanning electron microscopy analysis

For scanning electron microscopy (SEM) analysis, 2 ml samples of MED4 culture were collected at ZT 14 to ensure a large number of dividing cells [14]. Cell samples were first fixed with glutaraldehyde to a final concentration of 2% (w/v) in the dark and then filtered through a 0.2 μm Nucleopore Track-Etch Membrane (Whatman). Cells on the filter were further fixed in a 2% (w/v) glutaraldehyde/3% (w/v) paraformaldehyde/5% (w/v) sucrose solution in 0.1 M phosphate buffer. After the fixation, the filter was washed with 0.1 M phosphate buffer and dehydrated in an ethanol gradient of 50, 75, 95, 100 and 100% (v/v) ethanol for 15  min each. Samples were then dried and mounted on a copper plate with carbon tape. Cells on the filter were sputter-coated and observed on a JEOL-6390 scanning electron microscope (20 kV, Working distance = 8 mm) at the Materials Characterization and Preparation Facility in the Hong Kong University of Science and Technology.

### (f) Nanoparticle tracking analysis

Nanoparticle tracking analysis (NTA) was performed using either a NanoSight NS300 (488 nm laser) or a Particle Metrix ZetaView NTA (488 nm laser), and the data were analysed by the accompanying software, v. 3.4 for NS300 and v. 8.05.14_SP7 for ZetaView. Before injecting each sample, the sample chamber was washed with Milli-Q water, and real-time imaging confirmed that particles in the field ranged from 0 to 3. Samples were diluted to achieve particle concentrations ranging from 1 × 10^7^ to 9 × 10^8^ particles ml^−1^. The data were normalized to the culture used for purification. For each sample, at least three technical replicates were measured by pushing syringes and capturing different fields. For each independent experiment, data files were processed using identical settings. The setting ranges were based on the manufacturer’s guidelines and our observation (for Particle Metrix ZetaView: sensitivity 80, shutter 100, frame rate 30, minimum area 5, minimum brightness 25; for NanoSight NS300: camera level 10–15, detection threshold 4–8).

### (g) Coomassie blue staining

For Coomassie blue staining, the cell lysate sample was prepared from *ca* 6 ml *Prochlorococcus* MED4 culture (10^8^ cells ml^−1^) while the EV sample was purified from *ca* 500 ml culture. Cell pellets were harvested by centrifugation for 13 min at 12 000*g* at 4℃ (Himac CR22N, R20A2 rotor) and resuspended in phosphate-buffered saline (PBS) with 2 μg μl^−1^ proteinase inhibitor. The solution was ultrasonicated at 50% amplitude 6 times, 20 s in each time, followed by a brief centrifugation for 5 min at 14 000*g*. at 4℃. Cell pellets and EV pellets were incubated with sodium dodecyl sulfate–polyacrylamide gel electrophoresis (SDS-PAGE) loading buffer for 30 min at 55℃. The electrophoresis was performed a 14% SDS-PAGE gel. The protein gel was stained with Coomassie blue staining buffer at room temperature and washed with destaining buffer until clear protein bands appeared.

### (h) Mass spectrometry analysis

#### (i) Preparation of samples from extracellular vesicles and from cell lysates for mass spectrometry analysis

For proteomics analysis, each EV sample was purified from 500 ml *Prochlorococcus* MED4 culture. After flotation, the EV fraction (*ca* 200 μl) was diluted with 300 μl KOAc buffer and then pelleted by ultracentrifugation for 30 min at 120 000*g* at 4℃ (Hitachi CS150NX Micro Ultracentrifuge, rotor S120-AT3). EV pellets were resuspended in 0.1% RapiGest in TEAB buffer (50 mM triethylammonium bicarbonate). Urea was added, to a concentration of 4 M in a sample. The sample was then reduced with 10 mM Tris-(2-Carboxyethyl)phosphine (TCEP) at 37℃ for 1 h and alkylated with 20 mM iodoacetamide (IAA) at room temperature for 30 min in the dark. To digest proteins into peptides, sequencing-grade modified trypsin (Promega, number V511A) was added, and the reaction systems were incubated at 37℃ for 20 h. The pH of the digested samples was reduced to 2.5–3 by adding trifluoroacetic acid (TFA) to break down the RapiGest. Degraded RapiGest was removed after brief centrifugation for 10 min at 14 000*g* at 4℃. Sample supernatant was dried by speed vacuum concentrator. Samples were dissolved with 0.1% (v/v) TFA, desalted using an activated C18 spin column and dried again.

Each cell lysate sample was prepared from around 40 ml *Prochlorococcus* MED4 culture with concentration about 10^8^ cells ml^−1^. Cell pellets were harvested by centrifugation for 13 min at 12 000*g* at 4℃. Four hundred microlitres of PBS was used to resuspend cells before sonication. The cell debris was removed by centrifugation for 5 min at 14 000*g* at 4℃. Cell lysates were mixed with precooled acetone (1 : 4) and were incubated for 4 h at −20℃ to precipitate proteins from the cell lysate. White protein pellets appeared after centrifugation for 30 min at 14 000*g* at 4℃. After brief drying with air, protein pellets were resuspended in 0.1% (w/v) RapiGest in TEAB buffer. Samples from cell lysates were prepared for MS analysis using procedures identical to those performed to prepare EV samples.

#### (ii) Liquid chromatography–tandem mass spectrometry analysis

After vacuum drying, peptides were resuspended in buffer A (0.1% formic acid in water), and the concentration was estimated by measuring absorbance at 280 nm (NanoDrop 2000, Thermo Fisher Scientific, Waltham, MA, USA). All peptide samples were analysed using a Q Exactive HF-X hybrid quadrupole-Orbitrap mass spectrometer coupled to an EASY-nLC™ 1200 system (Thermo Fisher Scientific, Waltham, MA, USA). For each sample, 2 µl of peptide mixture was resolved using an analytical C18 column (250 mm, 75 µm, 3 µm; PepSep, Denmark) at a flow rate of 250 nl min^−1^ for 75 min. The mobile phase was a mixture of buffer A and buffer B (0.1% (v/v) formic acid in 80% (v/v) acetonitrile) with a changing gradient of buffer B as follows: 0–2 min at 3–7%; 2–52 min at 7–25%; 52–62 min at 25–44%; 62–70 min at 44–95%; and 70–75 min at 95%. MS recording was operated in the range of 350–1800 *m*/*z* with a mass resolution of 120 000. The positive ion mode was employed with the spray voltage at 2500 V, and a spray temperature of 320°C. The resolution of dd-MS^2^ was 30 000 with a 1 × 10^5^ AGC target. The maximum IT was set at 60 ms and the loop count was 12. The isolation window was 1.6 *m*/*z* and fixed first mass was 120.0 *m*/*z*.

#### (iii) Raw data processing

Raw MS data were processed using the Proteome Discoverer software v. 2.2 and proteins were identified by searching MS/MS spectra against a tryptic digest of *Prochlorococcus* MED4 database downloaded from UniProt. The MS/MS spectra were searched with carbamidomethyl/+57.021 Da (C) as static modification, as well as oxidation/+15.995 Da (M), acetyl/+42.011 Da (N-terminus) as dynamic modifications. A maximum of two missed cleavages was allowed, the minimum peptide length was set to six amino acids and the maximum peptide length was 144. The abundances of the protein were calculated by untargeted label-free quantification based on the precursor ion peaks across runs. Then, the quantified values were normalized based on the total peptide intensity of the samples to get the normalized abundances. Proteins identified by two or more unique peptides were used for analysis.

#### (iv) Bioinformatic analysis

Proteins with at least two valid values in all replicates were retained for bioinformatic analysis in the R programming environment (R v. 4.0.3 in RStudio v. 1.3.1093). The COG analysis was performed using the annotation information downloaded from UniProt. The proportions of proteins were calculated for differential expression analysis between cell lysate group and EV group, and Student’s *t*‐test was used to test for significant differences. The mean log_2_-fold changes in the EV group over the cell lysate group were calculated and plotted against the −log_10_ *p*‐value. GO analysis of predicted EV-enriched proteins was performed through Uniprot. As for DUSK and DAWN, proteins with at least two valid values in four replicates were kept, and missing values were imputed before using R package LIMMA for differential expression analysis.

## Data Availability

The list of proteins identified by mass spectrometry analysis can be found as supplementary material, available at [32]. The mass spectrometry data can be provided on request.
